# Link between alcohol consumption and myocardial infarction, stroke, and all-cause mortality among Swedish male automotive workers over 30 years

**DOI:** 10.1080/02813432.2026.2712981

**Published:** 2026-08-07

**Authors:** Hanna Asp, Lennart Dimberg

**Affiliations:** Department of Public Health and Community Medicine, University of Gothenburg, Gothenburg, Sweden

**Keywords:** Alcohol, all-cause mortality, myocardial infarction, stroke, longitudinal cohort study, Swedish males, Coeur study

## Abstract

**Introduction:**

The relationship between alcohol consumption and the incidence of myocardial infarction (MI), stroke and all-cause mortality exhibits significant regional variation.

**Objective:**

This prospective study aimed to investigate this association analyzing data from a cohort of Swedish men aged 45–50 years at baseline, evaluating subgroups over various time intervals, and compiling data on cardiovascular endpoints.

**Methods:**

The Coeur study, conducted between 1993 and 1995, included 973 middle-aged male employees out of a randomly selected sample of 1144 individuals from a Swedish automotive company. Participants were followed for 30 years using national health registers. The accumulated incidence of first-time stroke, first-time MI and all-cause mortality was calculated and adjusted for cardiovascular risk factors. Data analysis employed time-to-event methods (Kaplan–Meier’s estimates and hazard ratios) and measures of association (odds ratios and correlation analysis).

**Results:**

No statistically significant association was observed between alcohol consumption and stroke (*p* > 0.05). However, alcohol consumption was associated with lower odds of MI (OR = 0.57, 95% CI: 0.41–0.79) and a slight increase in survival time (HR = 0.997, 95% CI: 0.995–1.000). Alcohol consumers had a median survival advantage of 1.3 years compared to non-consumers. All beverage types showed a negative association with all-cause mortality (total alcohol: OR = 0.57; beer: OR = 0.68, 95% CI: 0.50–0.92; wine: OR = 0.55, 95% CI: 0.41–0.75). Conversely, beer consumption was linked to higher odds of MI (OR = 1.70, 95% CI: 1.05–2.75).

**Conclusions:**

While alcohol consumption in middle-aged men after 30 years follow-up is associated with increased survival time, beer correlates with a higher likelihood of MI.

## Introduction

Statistics Sweden reports that men’s life expectancy increased by approximately two years between the periods 2001–2010 and 2011–2020, largely attributable to a reduction in cardiovascular mortality [1]. Despite this progress, cardiovascular diseases continue to be a leading cause of death, highlighting the need for ongoing research into protective factors and underlying mechanisms to further enhance public health outcomes related to cardiovascular disease [2].

### Determinants of mortality and cardiovascular health

Key cardiovascular risk factors include high blood pressure, hyperlipidemia, smoking and body fat distribution [3]. The long-term impact of these and related factors on longevity is demonstrated in a Swedish longitudinal cohort study involving 855 men, which found that only 13% lived to age 90, with longevity included non-smoking, higher socioeconomic status, lower coffee consumption and low cholesterol levels [4]. In terms of socioeconomic factors, a study examining mortality among different occupational groups revealed that 12% of middle-aged men with low occupational status followed for 12 years died compared to 6% in the highest group [5]. Additionally, marriage appears to offer protective benefits; several studies indicate that unmarried individuals had 72% higher odds of mortality (OR = 1.72) and more than twice the odds of dying the first day after a coronary event compared to their married counterparts (OR = 2.14) [6,7].

### Alcohol and health

The relationship between alcohol consumption and cardiovascular health is complex and multifaceted. A well-known concept is ‘The French Paradox’, which emerged in the 1980s. This paradox refers to the epidemiological observation that France has relatively low coronary mortality despite high consumption of saturated fats, potentially explained by higher levels of wine consumption among the French population [8,9]. The protective effect of wine may be attributed to its flavonoid content, which is associated with increased levels of high-density lipoprotein cholesterol is one of the suggested explanations [2,9].

The cardiovascular impact of alcohol has been often illustrated by the J-shaped curve, which suggests that light to moderate drinking may lower morbidity compared to abstinence or heavy use. However, this model has faced criticism particularly regarding the ‘sick quitter’ hypothesis, which posits that former drinkers with poor health may be misclassified as abstainers potentially skewing results [10]. Despite this, some studies continue to investigate the proposed protective effects of alcohol, particularly by comparing different beverage types, although findings remain inconsistent. Moderate beer consumption has shown some protective effects in Swedish and international studies [11,12]. Conversely, other research emphasizes drinking frequency over type as a critical factor [13]. The global INTERHEART study confirmed a protective effect of alcohol on myocardial infarction (MI), though it noted considerable regional variation [14].

Conversely, even low alcohol intake may have negative effects on cardiovascular health. A Swedish study found a dose-dependent link between alcohol and diastolic dysfunction, showing increased odds even among low-level drinkers [15]. Furthermore, when considering alcohol’s effects beyond longevity and cardiovascular health, current national and international guidelines, including those from the World Health Organization, emphasize that no level of alcohol consumption is risk-free. Alcohol is linked to over 200 health conditions, including various cancers and cardiometabolic diseases, and it contributes to a significant number of deaths worldwide. The societal costs related to alcohol surpass any economic benefit derived from alcohol taxation [16,17]. In response to these concerns, several countries have implemented restrictive public health guidelines regarding alcohol consumption [9,16]. While Sweden lacks strict national alcohol limits, health care guidelines define risky consumption as more than 10 drinks per week or over four per occasion [18]. Overall, attitudes towards alcohol consumption are diverse, but recent perspective has shifted toward a more cautious approach. The cultural and historical use of alcohol challenge the establishments of new alcohol guidelines [19].

The Franco-Swedish Coeur study, initiated in 1993, aimed to investigate the potential French Paradox by comparing cohorts of 1000 middle-aged men from the automotive industry in both countries. By the study’s conclusion in 1998, the Framingham risk index was found to be similar between the two populations; however, the number of MIs recorded was too low to draw significant conclusions regarding country-specific differences [3].

Importantly, the Swedish cohort was accessible for long-term follow-up through national registries. The aim of this follow-up study is to evaluate the cardiovascular risks associated with alcohol consumption in the Swedish cohort after 30 years of monitoring.

## Methods

### Study population

This study builds on the 1993 Coeur Study [3]. Utilizing a prospective longitudinal design, it systematically integrates survey and clinical data with three decades of registry information from national health registers. The baseline data includes health and lifestyle information (alcohol use, blood pressure, lab tests, electrocardiograms and more) collected in 1993, along with data from a 1995 follow-up sub-sample of the original cohort [3,20].

Baseline data were collected from 1144 men (45–50 years) employed at Volvo (Gothenburg, Sweden). After excluding participants due to refusal, illness or cardiovascular treatment, 1000 individuals remained, of whom 973 were included in the present analysis (one outliner for alcohol consumption and 26 missing data were excluded). Additionally, 173 blood samples and 84 questionnaires from the 1995 sub-study, investigating alcohol consumption, meal patterns and cardiovascular risk, were incorporated.

**Figure 1.**
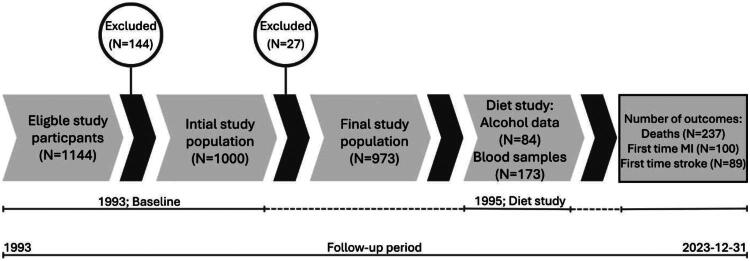
Timeline illustrating data sources and participant exclusions over time leading to the cohort of 973 middle-aged Swedish male employees at an automotive company in 1993. The diet study is a subsample of the original cohort. MI: myocardial infarction; *N*: number of participants.

Baseline registration initiated individual follow-up time (Figure 1).

**Figure 2.**
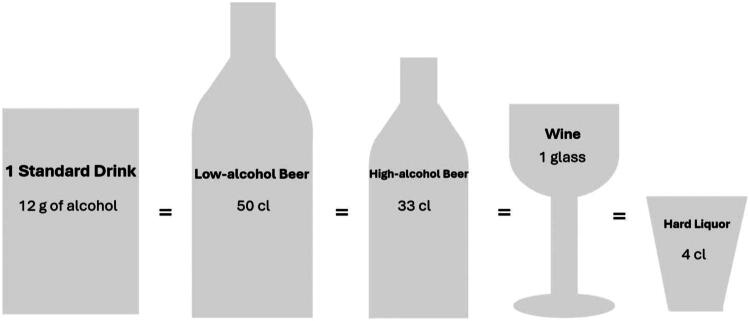
Illustration of one standard drink (12 g of pure alcohol), represented by equivalent amounts of various beverage types. g: grams of pure alcohol; cl: centiliter of specific beverage type; =: equals to.

The 973 participants were categorized by beverage type and total alcohol intake (g/week), calculated based on baseline self-reported consumption. Weekly intake was determined by multiplying the number of drinks per week by the estimated grams of pure alcohol per beverage type (beer: 6 g, wine: 14 g, hard liquor: 13 g) [3]. This results in quintiles, quartiles and binary classifications. Furthermore, based on Swedish healthcare guidelines, participants were grouped into risk (>120 g/week) and non-risk consumers (per-occasion risk could not be assessed due to missing data) [18]. The classification is presented in Table 1. One standard drink illustrated by quantity of beverage type is shown in Figure 2.

**Table 1.** Classification of beverage consumption by type and amount in the cohort consisting of 973 middle-aged Swedish male employees at an automotive company in 1993.

Alcohol (total)	Range (g/wk) 0–342	*N* 973
*Dichotomous groups*		
Non-alcohol drinkers	0	236
Alcohol drinkers	6–342	737
Non-risk group	0–119	886
Risk group	≥120	87
*Quintiles*		
Quintile 1	0	236
Quintile 2	6–27	154
Quintile 3	28–54	200
Quintile 4	55–84	192
Quintile 5	85–342	191
Beer	Range (g/wk)	*N*
	0–300	973
*Dichotomous groups*		
Non-beer drinkers	0	329
Beer drinkers	6–300	644
*Quartiles*		
Quartile 1	0	329
Quartile 2	6–12	202
Quartile 3	18–24	190
Quartile 4	30–300	252
Wine	Range (g/wk)	*N*
	0–294	972
*Dichotomous groups*		
Non-wine drinkers	0	521
Wine drinkers	14–294	451
*Quartiles*		
Quartile 1	0	521
Quartile 2	14–28	215
Quartile 3	42–84	174
Quartile 4	98–294	62

g/wk: grams per week; *N*: number of participants; risk: risk-drinking as determined by the Swedish guideline on alcohol consumption; ≥: greater than or equal to.

### The diet study

In 1995, a sub-sample of the original cohort included 90 middle aged men at low and 90 at high cardiovascular risk from Sweden. These men were invited to participate in interviews conducted by nurses to evaluate their meal habits, alcohol and food consumption. Table 2 illustrates the similarities with the original cohort. The analysis also included evaluations of blood-chemistry and responses from the questionnaires [20].

**Table 2.** Descriptive data were collected in the cohort of 973 middle-aged Swedish male employees at an automotive company in 1993 and subgroups participating in the 1995 diet study.

	All participants *N* = 973	Diet study: laboratory samples *N* = 173	Diet study: questionnaire *N* = 84
*Age (years), N (%)*			
45	67 (7)	14 (8)	2 (2)
46	195 (20)	35 (20)	11 (13)
47	170 (18)	38 (22)	21 (25)
48	215 (22)	42 (24)	30 (36)
49	211 (22)	34 (20)	20 (24)
50	115 (12)	10 (6)	0 (0)
*Outcomes, N (%)*			
All-cause mortality	237 (24)	48 (30)	33 (39)
Myocardial infarction	100 (10)	19 (11)	16 (19)
Stroke	89 (9)	11 (6)	7 (8)
*Alcohol consumption, median (IQR), g/wk*			
Total alcohol	40 (6–74)	33 (0–68)	24 (0–56)
Beer consumption	12 (0–30)	12 (0–24)	12 (0–24)
Wine consumption	0 (0–28)	0 (0–28)	0 (0–28)
*Lifestyle and cardiometabolic*			
Diabetes, *N* (%)	13 (1)	3 (2)	3 (4)
Exercise (<1 h/week), *N* (%)	305 (31)	55 (32)	27 (32)
Exercise (1–3 h/week), *N* (%)	490 (50)	80 (46)	39 (46)
Exercise (>3 h/week), *N* (%)	186 (19)	38 (22)	18 (21)
WHtR, median (IQR)	0.51 (0.48–0.55)	0.50 (0.46–0.54)	0.53 (0.50–0.57)
Systolic BP, median (IQR), mmHg	115 (107–125)	112 (104–123)	120 (112–132)
Current smokers, *N* (%)	272 (28)	57 (33)	56 (67)
Pack-years, median (IQR)	7 (0–20)	9 (0–23)	19 (6–28)
Clerical workers, *N* (%)	596 (61)	113 (65)	48 (57)
Manual workers, *N* (%)	377 (39)	60 (35)	36 (43)
Married or cohabitant, *N* (%)	742 (76)	128 (74)	60 (71)
Single, divorced or widowed, *N*	230 (24)	45 (26)	24 (29)
*Laboratory samples*			
Non-HDL Cholesterol, median	4.7 (4.0–5.4)	4.3 (3.5–4.9)	5.0 (4.5–5.3)
HDL Cholesterol, median (IQR),	1.2 (1.0–1.6)	1.2 (1.0–1.5)	1.0 (0.8–1.1)

BP: blood pressure; g/L: grams per litre; g/wk: grams per week; HDL: high-density lipoprotein; IQR: interquartile range; mmol/L: millimoles per litre; *N*: number of participants; WHtR: waist-to-height ratio; %: percentage, representing the proportion of observed participants in the cohorts defined by each column; >: greater than; =: equals to.

Missing data for the cohort of all participants (*N* = 973): wine consumption (*N* = 1, <0.01%), WHtR (*N* = 1, <0.01%), pack-years (*N* = 62, 6%), household status (*N* = 1, <0.01%) and non-HDL cholesterol (*N* = 67, 7%).

### Variables

Examinations and questionnaires were conducted during paid working hours by nurses at Volvo in Gothenburg and Trollhättan, Västra Götaland, Sweden. This forms the basis of the baseline data, and the factors used as covariates in this study; age (years), total alcohol consumption, beer consumption, wine consumption, hard liquor consumption (Figure 2), systolic blood pressure (a mean of three readings measured in supine position after 10 min rest), waist-to-height ratio (cm), diabetes status (yes or no, defined by fasting plasma glucose >7.0 mmol/L or self-reported diagnosis), pack-years of smoking (packages of 20 cigarettes multiplied with years of smoking), household status (categorized as married/cohabiting or single/divorced/widowed), occupational status (manual or clerical) and non-high-density lipoprotein cholesterol. Exercise categories were defined as follows: less than 1 h exercise per week, 1–3 h/week including longer walks and more than 3 h/week which includes individuals who are active as athletes (Table 2) [3].

Moreover, several laboratory blood samples have been incorporated in the present analysis including gamma-glutamyl transferase (μkat/L), high-density lipoprotein cholesterol (mmol/L), low-density lipoprotein cholesterol (mmol/L), triglycerides (mmol/L), plasma fibrinogen (g/L), serum cholesterol (measured in mmol/L) and plasma glucose (mmol/L). Fasting venous blood samples were collected, stored at −20 °C, and analysed at Sahlgrenska University hospital.

### Outcomes

Outcomes were established based on International Classification of Diseases (ICD) 9th and 10th edition and includes all-cause mortality (all ICD codes), first-time MI (ICD I21-I23) and first-time stroke (ICD I61-I64) [21]. Outcomes were identified via national health registers: the Swedish National Quality Register for Stroke Care [22], the Swedish National Death Register [23] and the Swedish National MI Registry [24] until and including 31 December 2023.

### Statistical analyses

Analyses were performed in SPSS (version 29-30) (Chicago, IL) with a significance threshold of *p* < 0.05 and 95% confidence intervals. Medians and interquartile ranges describe quantitative data, while percentages are used for dichotomous variables. Percentages in tables are rounded to whole numbers with values ending in 0.5 rounded up (totals may exceed 100%).

Statistical analyses include odds ratios and Spearman’s rho for associations, Kaplan–Meier’s analysis for cumulative incidence (censored at death and comparison via chi-square tests) and Cox regressions for survival. Covariates significant in univariates models are included in the multivariate model, excluding overlapping measures (beverage types in total alcohol models).

## Results

Statistically significant findings were observed for the primary outcomes of all-cause mortality and MI, whereas no significant results were found for stroke. Key characteristics of the study population are presented in Tables 2 and 3.

**Table 3.** Baseline characteristics for groups related to self-reported alcohol consumption in the cohort of 973 middle-aged Swedish male employees at an automotive company in 1993.

	Alcohol consumption, median (IQR), g/wk	HDL-cholesterol, median (IQR), mmol/L	Occupational status, clerical workers, *n* (%)	Household status, married or cohabitant, *n* (%)
Non-alcohol drinkers, *n* = 236 (0 g/wk)	0 (0–0)	1.1 (0.99–1.3)	86 (36%)	162 (69%)
Alcohol drinkers, *n* = 737 (6–342 g/wk)	56 (30–86)	1.2 (1.0–1.4)	510 (69%)	580 (79%)
Non-risk group, *n* = 886 (0–119 g/wk)	33 (0–65)	1.2 (1.0–1.4)	542 (61%)	670 (76%)
Risk group, *n* = 87 (≥120 g/wk)	168 (137–204)	1.3 (1.0–1.5)	54 (62%)	72 (83%)
Non-beer drinkers, *n* = 329 (0 g/wk)	0 (0–26)	1.2 (1.0–1.3)	143 (44%)	236 (72%)
Beer drinkers, *n* = 644 (1–300 g/wk)	57 (20–88)	1.2 (1.0–1.4)	453 (70%)	506 (79%)
Non-wine drinkers, *n* = 521 (0 g/wk)	12 (0–36)	1.1 (1.0–1.3)	248 (48%)	361 (69%)
Wine drinkers, *n* = 451 (1–294 g/wk)	70 (48–104)	1.2 (1.1–1.4)	348 (77%)	380 (84%)
Q1 (total alcohol), *n* = 236, 0 g/wk	0 (0-0)	1.1 (1.0–1.3)	86 (36%)	162 (69%)
Q2 (total alcohol), *n* = 154, 6–27 g/wk	16 (12–24)	1.1 (0.95–1.3)	104 (68%)	116 (76%)
Q3 (total alcohol), *n* = 200, 28–54 g/wk	40 (33–45)	1.2 (1.0–1.4)	135 (68%)	151 (76%)
Q4 (total alcohol), *n* = 192, 55–84 g/wk	40 (33–45)	1.2 (1.1–1.4)	137 (71%)	158 (82%)
Q5 (total alcohol), *n* = 191, 85–342 g/wk	67 (60–75)	1.2 (1.0–1.5)	134 (70%)	155 (81%)
Q1 (beer), *n* = 329, 0 g/wk	0 (0–0)	1.2 (1.0–1.3)	143 (44%)	236 (72%)
Q2 (beer), *n* = 202, 6–12 g/wk	34 (12–51)	1.2 (1.0–1.3)	155 (77%)	155 (77%)
Q3 (beer), *n* = 190, 18–24 g/wk	52 (30–73)	1.2 (1.1–1.4)	133 (70%)	158 (83%)
Q4 (beer), *n* = 252, 30–300 g/wk	86 (58–130)	1.3 (1.0–1.4)	165 (66%)	193 (77%)
Q1 (wine), *n* = 521, 0 g/wk	12 (0–36)	1.1 (0.99–1.3)	248 (48%)	361 (69%)
Q2 (wine), *n* = 215, 14–28 g/wk	51 (34–65)	1.2 (1.0–1.4)	168 (78%)	181 (84%)
Q3 (wine), *n* = 174, 42–84 g/wk	85 (67–111)	1.3 (1.1–1.5)	135 (78%)	146 (84%)
Q4 (wine), *n* = 62, 98–294 g/wk	161 (111–217)	1.3 (1.1–1.6)	45 (73%)	53 (86%)

The number of individuals who are manual workers is determined by subtracting the number of clerical workers from the total number of individuals in the cohort, as indicated in the column to the left. The number of individuals who are single, divorced or widowed is determined by subtracting the number of married/cohabiting individuals from the total number of individuals in the cohort, as indicated in the column to the left.

The distribution of mortality by household status reveals that single, divorced and widowed workers experienced nearly twice the mortality rate compared to their married or cohabitant counterparts.

**Figure 3.**
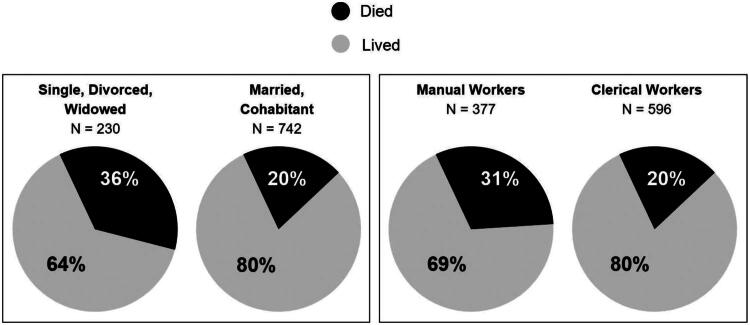
Distribution of mortality by household and occupational status of the cohort of 973 Swedish male industrial workers in 1993, followed for 30 years. *N*: number of participant; %: percentage, representing mortality proportions; =: equals.

Additionally, manual workers face an almost 59% higher mortality rate than clerical workers (Figure 3).

**Figure 4.**
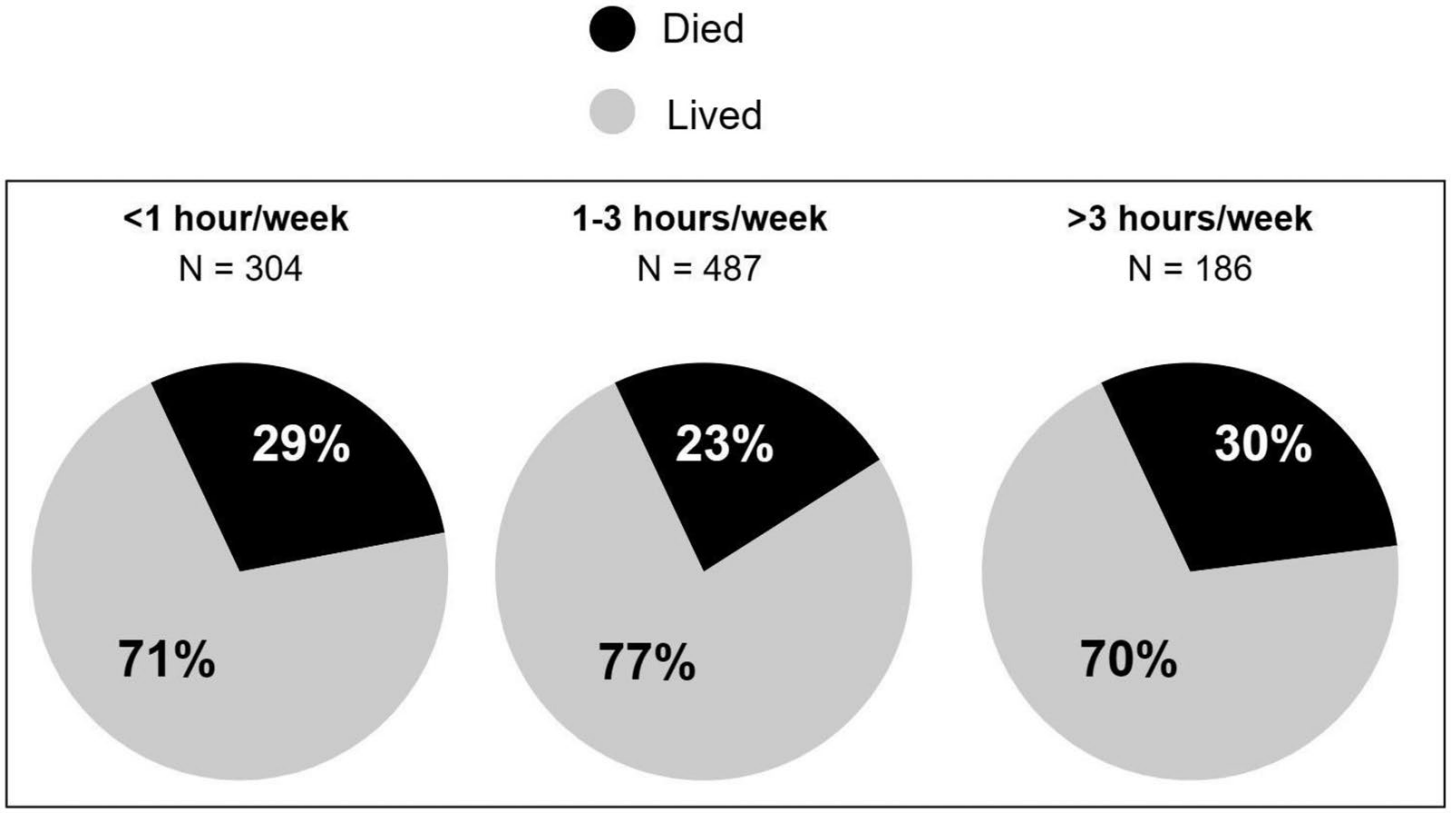
Distribution of mortality by level of exercise at study start: less than 1 h/week, 1–3 h/week and more than 3 h/week of the cohort of 973 Swedish male industrial workers in 1993, followed for 30 years. *N*: number of participant; %: percentage, representing mortality proportions; =: equals.

Furthermore, mortality distributions based on exercise levels indicate a lower proportion of deaths among individuals engaging in the middle level of exercise (see Figure 4).

Evaluating the descriptive data between the sub-groups showed notable differences in characteristics as following: alcohol and wine drinkers had slightly higher high-density lipoprotein cholesterol levels than non-drinkers, while no difference was seen for beer. Drinking groups also had higher proportions of clerical workers and married/cohabiting individuals compared to abstainers (Table 3).

### Correlations by type of alcohol

Spearman’s rho analysis showed significant positive correlations between high-density lipoprotein levels (from 1993) and self-reported alcohol intake from the same year, aligning with the descriptive data for wine and total-alcohol drinkers. To evaluate the validity of self-reported alcohol, several analyses were conducted. No correlation was found between self-reported alcohol use and gamma-glutamyl transferase in the 1995 sub-cohort. However, a moderate positive correlation (*R* = 0.58) was observed between self-reported alcohol consumption at baseline (1993) and data from the more detailed diet study conducted in a subgroup assessed in 1995 (Table 4).

**Table 4.** Correlation analyses measured with Spearman’s rho of the data from the cohort of 973 middle-aged Swedish male employees at an automotive company in 1993, and a subgroup participating in the 1995 diet study.

Laboratory test	Alcohol	*N*	Spearman’s rho	95% CI
HDL-cholesterol (1993), mmol/L	Total alcohol (1993), g/wk	906	0.19	0.123–0.252
HDL-cholesterol (1993), mmol/L	Wine (1993), g/wk	905	0.17	0.108–0.238
HDL-cholesterol (1993), mmol/L	Beer (1993), g/wk	906	0.14	0.075–0.206
HDL-cholesterol (1995), mmol/L	Total alcohol (1993), g/wk	173	0.27	0.116–0.402
HDL-cholesterol (1995), mmol/L	Total alcohol (1995), g/wk	80	0.094	−0.135 to 0.331
HDL-cholesterol (1995), mmol/L	Wine (1993), g/wk	173	0.29	0.142–0.424
HDL-cholesterol (1995), mmol/L	Beer (1993), g/wk	173	0.23	0.076–0.368
GGT (1995), µkat/L	Total alcohol (1993), g/wk	173	0.14	−0.013 to 0.288
GGT (1995), µkat/L	Total alcohol (1995), g/wk	84	0.19	−0.036 to 0.401
Total alcohol (1993), g/wk	Total alcohol (1995), g/wk	84	0.58	0.422–0.715

CI: confidence interval; g/wk: grams per week; GGT: gamma-glutamyl transferase; HDL: high-density lipoprotein; mmol/L: millimoles per litre; *n*: number of participants; *p* value: probability value; %: percent; µkat/L: microkatal per litre.

### Odds ratios of cardiovascular outcomes by type of beverage

The odds ratios conducted in this paper showed higher mortality rates in groups with lower alcohol intake. The data indicate an inverse relationship between alcohol consumption and mortality and suggest that the type of alcoholic beverage consumed may not be the primary determinant of mortality outcomes. Instead, total alcohol and wine intake appears to be more significant factors (Tables 5–7).

**Table 5.** Incidence and odds ratios of all-cause mortality, first-time myocardial infarction and first-time stroke in groups related to different consumption patterns of total alcohol consumption (g/week) in the cohort of 973 middle-aged Swedish male employees at an automotive company in 1993, followed for 30 years.

	Non-alcohol drinkers, *n* = 236 (0 g/wk)	Alcohol drinkers, *n* = 737 (6–342 g/wk)	Q1 (total alcohol), *n* = 236, 0 g/wk	Q2 (total alcohol), *n* = 154, 6–27 g/wk	Q3 (total alcohol), *n* = 200, 28–54 g/wk	Q4 (total alcohol), *n* = 192, 55–84 g/wk	Q5 (total alcohol), *n* = 191, 85–342 g/wk	Non-risk group, *n* = 886 (0–119 g/wk)	Risk group, *n* = 87 (≥120 g/wk)
All-cause mortality (%)	77 (33%)	160 (22%)	77 (33%)	33 (21%)	45 (23%)	40 (21%)	42 (22%)	218 (25)	19 (22)
OR (95% CI), *p* value	Ref. 1.00	0.57 (0.41–0.79), **<0.001**	Ref. 1.00	0.56 (0.35–0.90), **0.017**	0.60 (0.39–0.92), **0.019**	0.54 (0.35–0.85), **0.007**	0.58 (0.38–0.90), **0.015**	Ref. 1.00	0.86 (0.50–1.46), 0.567
Myocardial infarction (%)	19 (8%)	81 (11%)	19 (9%)	25 (16%)	17 (9%)	16 (8%)	23 (12%)	89 (10.0)	11 (12.6)
OR (95% CI), *p* value	Ref. 1.00	1.41 (0.84–2.4), 0.197	Ref. 1.00	2.2 (1.2–4.2), **0.014**	1.1 (0.54–2.1), 0.865	1.0 (0.52–2.1), 0.916	1.6 (0.82–3.0), 0.171	Ref. 1.00	1.3 (0.66–2.5), 0.447
Stroke (%)	20 (8%)	69 (9%)	20 (8%)	16 (10%)	16 (8%)	20 (10%)	17 (9%)	80 (9%)	9 (10%)
OR (95% CI), *p* value	Ref. 1.00	1.1 (0.66–1.9), 0.681	Ref. 1.00	1.3 (0.63–2.5), 0.524	0.94 (0.47–1.9), 0.858	1.3 (0.66–2.4), 0.493	1.1 (0.54–2.1), 0.876	Ref. 1.00	1.2 (0.56–2.4), 0.685

CI: confidence interval; g/wk: grams per week; *N*: number of participants; OR: odds ratio; *p* value: probability value; *Q*: quintile (total alcohol consumption); risk: risk-drinking as determined by the Swedish guideline on alcohol consumption; %: percentage, representing the proportion of observed participants in the cohorts defined by each column; <: less than; =: equals; ≥: equals to or greater than. Bolded p-values mean statistically significant.

**Table 6.** Incidence and odds ratios of all-cause mortality, first-time myocardial infarction and first-time stroke in groups related to different wine consumption patterns (g/week) in the cohort of 973 middle-aged Swedish male employees at an automotive company in 1993, followed for 30 years.

	Non-wine drinkers, *n* = 521 (0 g/wk)	Wine drinkers, *n* = 451 (1–294 g/wk)	Q1 (wine), *n* = 521, 0 g/wk	Q2 (wine), *n* = 215, 14–28 g/wk	Q3 (wine), *n* = 174, 42–84 g/wk	Q4 (wine), *n* = 62, 98–294 g/wk
All-cause mortality (%)	153 (29%)	84 (19%)	153 (29%)	41 (19%)	32 (18%)	11 (18%)
OR (95% CI), *p* value	Ref. 1.00	0.55 (0.41–0.75), **<0.001**	Ref. 1.00	0.57 (0.38–0.84), **0.004**	0.542 (0.354–0.831), **0.005**	0.519 (0.263–1.022), **0.06**
Myocardial infarction (%)	58 (11%)	42 (9%)	58 (11%)	17 (8%)	21 (12%)	4 (7%)
OR (95% CI), *p* value	Ref. 1.00	0.82 (0.54–1.2), 0.352	Ref. 1.00	0.69 (0.39–1.2), 0.191	1.1 (0.64–1.9), 0.736	0.55 (0.19–1.6), 0.27
Stroke (%)	48 (9%)	41 (9%)	48 (9%)	19 (9%)	18 (10%)	4 (7%)
OR (95% CI), *p* value	Ref. 1.00	0.87 (0.64–1.2), 0.380	Ref. 1.00	0.90 (0.54–1.2), 0.270	1.1 (0.72–1.6), 0.723	0.60 (0.28–1.2), 0.164

CI: confidence interval; g/wk: grams per week; *N*: number of participants; OR: odds ratio; *p* value: probability value; *Q*: quartile (wine consumption); %: percentage, representing the proportion of observed participants in the cohorts defined by each column; <: less than; =: equal. Bolded p-values mean statistically significant.

**Table 7.** Incidence and odds ratios of all-cause mortality, first-time myocardial infarction and first-time stroke in groups divided by quartiles of beer consumption (g/week) in the cohort of 973 middle-aged Swedish male employees at an automotive company in 1993, followed for 30 years.

	Non-beer drinkers, *n* = 329 (0 g/wk)	Beer drinkers, *n* = 644 (1–300 g/wk)	Q1 (beer), *n* = 329, 0 g/wk	Q2 (beer), *n* = 202, 6–12 g/wk	Q3 (beer), *n* = 190, 18–24 g/wk	Q4 (beer), *n* = 252, 30–300 g/wk
All-cause mortality (%)	96 (29%)	141 (22%)	96 (29%)	41 (20%)	41 (22%)	59 (23%)
OR (95% CI), *p* value	Ref. 1.00	0.68 (0.50–0.92), **0.013**	Ref. 1.00	0.62 (0.41–0.94), **0.024**	0.67 (0.44–1.0), 0.06	0.74 (0.51–1.1), 0.10
Myocardial infarction (%)	24 (7%)	76 (12%)	24 (7%)	24 (12%)	21 (11%)	31 (12%)
OR (95% CI), *p* value	Ref. 1.00	1.70 (1.1–2.8), **0.030**	Ref. 1.00	1.7 (0.95–3.1), 0.08	1.6 (0.85–2.9), 0.10	1.8 (1.0–3.1), **0.043**
Stroke (%)	29 (9%)	60 (9%)	29 (9%)	18 (9%)	19 (10%)	23 (9%)
OR (95% CI), *p* value	Ref. 1.00	1.1 (0.67–1.7), 0.797	Ref. 1.00	1.0 (0.55–1.9), 0.970	1.1 (0.62–2.1), 0.654	1.0 (0.59–1.8), 0.896

CI: confidence interval; g/wk: grams per week; *N*: number of participants; OR: odds ratio; *p* value: probability value; Qt: quartile (beer consumption); %: percentage, representing the proportion of observed participants in the cohorts defined by each column; =: equals.Bolded p-values mean statistically significant.

Regarding MI, higher rates of MI were observed in groups with higher alcohol consumption (Tables 5 and 6). Statistically significant odds ratios were identified across quintiles of total alcohol intake, quartiles of beer consumption, and in comparisons between beer drinkers and non-beer drinkers (Tables 5–7).

### Hazard rations of cardiovascular outcomes by types of beverage

When incorporating the factor of time, the hazard ratios and Kaplan–Meier analyses were consistent with the odds ratio analyses. When adjusted for covariates, total alcohol showed a hazard ratio of 0.997 (95% CI: 0.995–1.000). Even wine showed a hazard ratio indicating longer survival time (hazard ratio = 0.993 (95% CI: 0.988–0.988)), this was however only significant in the unadjusted model (Table 8).

**Table 8.** Unadjusted and adjusted hazard ratios for all-cause mortality associated with total alcohol, beer and wine consumption in the cohort of 973 middle-aged Swedish male employees at an automotive company in 1993, followed for 30 years.

Covariate	HR	95% CI	*p* Value	HR, adj.	95% CI, adj.	*p* Value, adj.
Total alcohol, g/wk	0.998	0.995–1.000	0.09	0.997	0.995–1.000	**0.045**
Beer, g/wk	0.999	0.994–1.001	0.6	0.998	0.993–1.003	0.4
Wine, g/wk	0.993	0.988–0.998	**0.005**	0.995	0.990–1.000	0.05

Adj: adjusted; CI: confidence interval; g/wk: grams per week; HR: hazard ratio; *p* value: probability value; %: percentage. Bolded p-values mean statistically significant.

Cox regression analyses were adjusted for the following covariates: age at baseline, systolic blood pressure, waist-to-height ratio, pack-years of smoking, diabetes status, occupational status and household status. The Cox regression model for beer consumption was additionally adjusted for wine intake.

Hazard ratio for beer drinkers was however slightly increased for MI (Table 9).

**Table 9.** Unadjusted and adjusted hazard ratios for first-time myocardial infarction associated with total alcohol, beer and wine consumption in the cohort of 973 middle-aged Swedish male employees at an automotive company in 1993, followed for 30 years.

Covariate	HR	95% CI	*p* Value	HR, adj.	95% CI, adj.	*p* Value, adj.
Total alcohol, g/wk	1.001	0.998–1.005	0.529	1.002	0.998–1.005	0.308
Beer, g/wk	1.005	1.000–1.011	0.050	1.005	1.000–1.010	**0.049**
Wine, g/wk	0.999	0.993–1.005	0.819	1.001	0.995– 1.007	0.766

Adj: adjusted; CI: confidence interval; g/wk: grams per week; HR: hazard ratio; *p* value: probability value; %: percentage.

Cox regression analyses were adjusted for the following covariates: systolic blood pressure, waist-to-height ratio, pack-years of smoking, non-HDL cholesterol and occupational status. The Cox regression model for wine consumption was additionally adjusted for beer intake.

The hazard ratios for first time stroke were not significant (Table 10).

**Table 10.** Unadjusted and adjusted hazard ratios for first-time stroke associated with total alcohol, beer and wine consumption in the cohort of 973 middle-aged Swedish male employees at an automotive company in 1993, followed for 30 years.

Covariate	HR	95% CI	*p* Value	HR, adjusted	95% CI, adjusted	*p* Value, adjusted
Total alcohol, g/wk	1.001	0.998–1.005	0.514	1.000	0.996–1.004	0.946
Beer, g/wk	1.002	0.996–1.009	0.457	0.999	0.992–1.006	0.807
Wine, g/wk	1.001	0.994–1.007	0.859	1.001	0.995–1.008	0.670

Adj: adjusted; CI: confidence interval; g/wk: grams per week; HR: hazard ratio; *p* value: probability value; %: percentage.

Cox regression analyses were adjusted for the following covariates: systolic blood pressure, waist-to-height ratio, pack-years of smoking, non-HDL cholesterol and occupational status. The Cox regression model for wine consumption was additionally adjusted for beer intake.

### Kaplan–Meier’s survival analyses of cardiovascular outcomes by types of beverage

**Figure 5.**
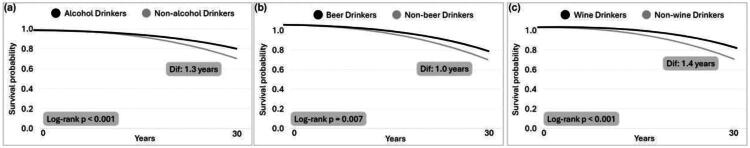
Distribution of mortality over time for subgroups of alcohol users in the cohort of 973 middle-aged Swedish employees at an automotive company in 1993, followed for 30 years. Time-to-event analyses were conducted using the Kaplan–Meier estimator, with *p* values calculated using the log-rank test*. p* value: probability value; <: less than; =: equals to.

The Kaplan–Meier curves showed a statistically longer mean time to survival for all groups of alcohol drinkers (Figure 5).

Beer-drinkers were the only group with a statistically significant difference in the Kaplan–Meier analyses for MI, where beer-drinkers had a MI 0.6 years before non-beer drinkers (Table 11).

**Table 11.** Kaplan–Meier’s survival analyses of time to first-time myocardial infarction in the cohort of 973 middle-aged Swedish male employees at an automotive company in 1993, followed for 30 years.

Myocardial infarction	Non-alcohol drinkers/alcohol drinkers	Non-risk group/risk group	Non-beer drinkers/beer drinkers	Non-wine drinkers/wine drinkers
K–M estimated mean time to event (years)	29.0/28.7	28.8/28.1	29.1/28.5	28.5/28.9
*χ*^2^ (df), *p* value	1.7 (1), 0.197	0.6 (1), 0.431	4.8 (1), **0.029**	0.9 (1), 0.336

CI: confidence interval; df: degrees of freedom; K–M: Kaplan–Meier; *p* value: probability value; *χ*^2^: chi-square. Bolded p-values mean statistically significant.

Time-to-event analyses were conducted using the Kaplan–Meier estimator, with *p* values calculated using the log-rank test.

Time to death was 1.3 years longer for alcohol drinkers, and 1.4 years longer for wine drinkers and 1 year longer for beer drinkers (Table 12).

**Table 12.** Kaplan–Meier’s survival analyses of time to death in the cohort of 973 middle-aged Swedish male employees at an automotive company in 1993, followed for 30 years.

All-cause mortality	Non-alcohol drinkers/alcohol drinkers	Non-risk group/risk group	Non-beer drinkers/beer drinkers	Non-wine drinkers/wine drinkers
K–M estimated mean time to event (years)	26.8/28.1	27.7/27.9	27.1/28.1	27.1/28.5
*χ*^2^ (df), *p* value	12.6 (1), **<0.001**	0.3 (1), 0.6	7.2 (1), **0.007**	15.8 (1), **<0.001**

CI: confidence interval; df: degrees of freedom; K–M: Kaplan–Meier; *p* value: probability value; *χ*^2^, chi-square; <: less than.Bolded p-values mean statistically significant.

Time-to-event analyses were conducted using the Kaplan–Meier estimator, with *p* values calculated using the log-rank test.

The time to MI events did not show significant differences for total alcohol and wine consumers. However, beer drinkers experienced their first on average MI 0.6 years earlier than non-beer drinkers (Table 13).

**Table 13.** Kaplan–Meier’s survival analyses of time to first-time myocardial infarction in the cohort of 973 middle-aged Swedish male employees at an automotive company in 1993, followed for 30 years.

Myocardial infarction	Non-alcohol drinkers/alcohol drinkers	Non-risk group/risk group	Non-beer drinkers/beer drinkers	Non-wine drinkers/wine drinkers
K–M estimated mean time to event (years)	29.0/28.7	28.8/28.1	29.1/28.5	28.5/28.9
*χ*^2^ (df), *p* value	1.7 (1), 0.197	0.6 (1), 0.431	4.8 (1), **0.029**	0.9 (1), 0.336

CI: confidence interval; df: degrees of freedom; K–M: Kaplan–Meier; *p* value: probability value; *χ*^2^: chi-square. Bolded p-values mean statistically significant.

Time-to-event analyses were conducted using the Kaplan–Meier estimator, with *p* values calculated using the log-rank test.

No significant differences in the time to first stroke were observed for any type of alcoholic beverage (Table 14).

**Table 14.** Kaplan–Meier’s survival analyses of time to first-time stroke by group of beverage drinkers in the cohort of 973 middle-aged Swedish male employees at an automotive company in 1993, followed for 30 years.

Stroke	Non-alcohol drinkers/alcohol drinkers	Non-risk group/risk group	Non-beer drinkers/beer drinkers	Non-wine drinkers/wine drinkers
K–M estimated mean time to event (years)	29.2/29.1	29.2/29.0	29.2/29.1	29.1/29.2
*χ*^2^ (df), *p* value	0.2 (1), 0.678	0.1 (1), 0.700	0.06 (1), 0.802	0.008 (1), 0.929

CI: confidence interval; df: degrees of freedom; K–M: Kaplan–Meier; *p* value: probability value; *χ*^2^: chi-square.

Time-to-event analyses were conducted using the Kaplan–Meier estimator, with *p* values calculated using the log-rank test.

See the main findings summarized in Table 15.

**Table 15.** Summary of results in the cohort of 973 middle-aged Swedish male employees at an automotive company in 1993, followed for 30 years.

Groups compared	Life expectancy	Myocardial infarction	Household status (married, cohabitants)	Occupational status (clerical workers)	HDL
Alcohol drinkers	+	+	+	+	+
Non-alcohol	−	−	−	−	−
Beer drinkers	+	+	+	+	**=**
Non-beer drinkers	−	−	−	−	**=**
Wine drinkers	+	*NS*	+	+	+
Non-wine drinkers	−	*NS*	−	−	−

NS: non-significant; HDL: high-density lipoprotein; (+) increased; (−) decreased; (=) same value.

The symbols + and – in the respective columns indicate the following: life expectancy: (+) increased life- expectancy compared to the opposing group, (−) decreased life expectancy. Myocardial infarction: (+) increased likelihood of myocardial infarction compared to the opposing group, (−) decreased likelihood. Household status: (+) higher proportion of married/cohabiting individuals compared to the opposing group, (−) lower proportion. Occupational status: (+) higher proportion of clerical workers compared to the opposing group, (−) lower proportion. HDL: (+) higher median value of HDL compared to the opposing group, (−) lower median value.

## Discussion

### The role of beverage type, quantity of alcohol, and socioeconomic confounding

This study found that alcohol consumption, regardless of quantity of beverage type, was associated with longer survival time. However, beer consumption was linked to an increased likelihood of MI. Additionally, both marital and occupational status was linked to an increased likelihood of MI.

The J-shaped curve hypothesis looks more like an L-shape in this population [25–27]. Similar to findings in other studies, no significant differences in mortality were observed across various levels of alcohol consumption or beverage types [13]. The results regarding MI diverge from existing literature, which generally suggests that moderate beer consumption as the most frequently consumed beverage reduces cardiovascular risk [11,13]. Notably, wine shows protective benefits in univariate models, but this effect disappears in multivariate analysis, indicating potential confounding by factors like marital and occupational status. A similar pattern emerges for beer, though in the opposite direction, suggesting that its apparent MI risk may reflect associated harmful factors rather than the beverage itself.

The inconclusive high density lipoprotein results could be influenced by confounding factors such as smoking (lowers HDL) and physical activity (raises HDL) [28]. In this cohort, approximately, 25% were smokers, and only 19% met recommended activity levels [29]. The findings related to all-cause mortality suggest that being married and having clerical employment may offer protective effects, consistent with previous literature. This may be explained by enhanced social support, quicker access to healthcare, and improved recovery following acute cardiac events [5,6,30,31].

However, despite beer drinkers being more frequently married and employed in clerical roles, they exhibited higher odds of MI, reinforcing the notion that unmeasured harmful factors associated with beer consumption may contribute to this association.

### Are alcohol consumption habits constant over time?

The Central Association for Alcohol and Drug information, which regularly survey random samples of Swedes, shows that the differences between young men (17–29 years) and older individuals (65–85 years) in Sweden since 2004 have nearly disappeared, partly because older adults have increased their consumption by one-third. Older men drink more frequently and in a more spread-out manner than younger men, making their habits less focused on intoxication [32].

Data were sourced from the UK population-based cohort: the Whitehall II Cohort which, included 6895 male British civil servants aged 35.55 in 1985. Alcohol consumption was self-reported at four different time points over a span of 20 years. The most common alcohol consumption trajectories were ‘stable moderate’ (40%) and mostly moderate (25%), accounting for 65% of the participants over the study period [33].

### Strengths and weaknesses

This investigation, focusing on a specific cohort of middle-aged Swedish men, employed in the automotive industry, has several strengths, including random sampling, high participation rates, standardized assessments, exclusion of individuals on long-term sick leave, and an extensive follow-up using validated methods and national registers.

However, the generalizability of this study is limited due to its specific focus on middle-aged Swedish men with stable employment, thereby excluding women and other demographic groups. The registered habits were based on baseline measurements, which may have varied during the follow-up period.

Unmeasured lifestyle factors, such as diet and cultural context, could also have influenced the results.

Moreover, the low representation of high-risk drinkers in the sample [34] and the reliance on self-reported alcohol intake may have compromised the validity of the findings. This could potentially account for the null result concerning gamma-glutamyl transferase [31]. The reliability of the alcohol-related questions posed in 1993 and 1995 was supported by a significant correlation (*R* = 0.58), outcome data from national registers may have overlooked non-hospitalized or silent events, although fatal outcomes were well documented [35,36].

## Conclusions

The findings of this study suggest a potential survival benefit associated with alcohol consumption, alongside a negative impact of beer specifically related to MI outcomes. However, these results must be interpreted cautiously due to limitations in generalizability and sample size. Further research involving larger and more ethnically diverse populations, as well as a more comprehensive evaluation of drinking patterns and lifestyle factors, is necessary. Only then can definitive conclusions be drawn, and current public health recommendations for specific populations such as the one studied, potentially revised.

## Data Availability

Data generated during the study are available from the corresponding author on reasonable request.
